# The Role of Climate in the Epidemiology of Melioidosis

**DOI:** 10.1007/s40475-017-0124-4

**Published:** 2017-08-19

**Authors:** Adam J. Merritt, Timothy J. J. Inglis

**Affiliations:** 1 Department of Microbiology, PathWest Laboratory Medicine Western Australia, PP Building, QEII Medical Centre, Hospital Avenue, Nedlands, WA 6009 Australia; 2 School of Biomedical Sciences, Faculty of Health and Medical Sciences, The University of Western Australia (M504), 35 Stirling Highway, Crawley, WA 6009 Australia

**Keywords:** *Burkholderia pseudomallei*, Melioidosis, Climate, Epidemiology

## Abstract

**Purpose of Review:**

Melioidosis epidemiology is susceptible to climate change through direct and indirect effects on human encounter with the causative agent, *Burkholderia pseudomallei*. This review describes the current depth of knowledge and recent advances in the understanding of this relationship and applies it to observations of melioidosis in Western Australia.

**Recent Findings:**

High maximum rainfall and dense cloud cover have been shown to predict environmental presence of *B. pseudomallei* and cases of melioidosis, probably through correspondingly high moisture levels in *B. pseudomallei*-receptive soils. Increased melioidosis cases have been observed following storms in Taiwan and cyclones in the Australian Northern Territory and strengthen the association between melioidosis and extreme weather events. Indirect weather effects contribute to bacterial exposure through mechanisms such as increasing *B. pseudomallei* output from water seeps after heavy rain or localised flooding. Climate and weather have been directly implicated in dissemination of *B. pseudomallei* and cases of melioidosis in several notable events in Western Australia. Over a 10-year surveillance period, the cases that lay in the path of a tropical cyclone co-located with cyclone systems that repeatedly crossed the Western Australian coast. Cyclone-associated cases were caused by different *B. pseudomallei* MLST genotypes, arguing against airborne dissemination from a common source.

**Summary:**

Predicted increases in temperature, changes in global precipitation patterns and an increased incidence of extreme weather events are expected to change melioidosis epidemiology. Further studies of the physical geographic drivers of melioidosis will deepen understanding of the impact of climate on melioidosis.

## Introduction

Current dogma considers melioidosis a disease of the tropics. Melioidosis investigators, these authors included, consider the main risks to be residence in or travel to a melioidosis-endemic region. However, as new countries and regions are added to the melioidosis prevalence zone, sporadic cases occur in the sub-tropics, or small clusters of infection occur sporadically in temperate areas, the epidemiological generalisation that hot plus wet equals melioidosis must adapt to reflect these observations. The clinical aspects of melioidosis and the characteristics of *Burkholderia pseudomallei*, its bacterial causative agent, have been amply covered elsewhere [1–5]. In this review, we explore the current role that climate has in the epidemiology of melioidosis, how it has shaped melioidosis in Western Australia and consider where climate change might lead.

## Climate

The association of melioidosis with rainfall has been long established [6–11]. In 2003, Currie and Jacups found a correlation between intense rainfall and multiple forms of melioidosis over a 12-year study period [9]. Kaestli et al., extended the study period to 23 years and strengthened the previously observed correlation [12], while recognising the predictive importance of cloud cover to the occurrence of melioidosis. They proposed that levels of cloud cover correspond to the levels of soil moisture that provide suitable conditions for bacterial survival. They also noted that dense cloud cover would also provide protection from bactericidal UV wavelengths in sunlight citing work we previously conducted into the UV sensitivity *of B. pseudomallei* [13]. In the largest global compilation of melioidosis cases to date, Limmathurosakul et al., established a predictive association between a high maximum rainfall (but not minimum or mean), athrosol and acrisol soil types (soil formed or heavily modified by long-term human activity and acidic, clay-rich soil, respectively) and to a lesser extent salinity and gravel content and the presence of *B. pseudomallei*. They produced a predictive global map of *B. pseudomallei* suitability, disease incidence and mortality of melioidosis using a combination of this data with demographic, geopolitical, health care and environmental data. A matter of concern was the observation that much of South America, Africa, Middle East and East Asia featured in the proposed melioidosis-endemic zones despite either a complete absence of confirmed cases or only occasional sporadic case notification. The melioidosis-suitability heat map of Australia showed a potential endemic area further south than conventionally accepted. Additionally, atypical intense rainfall in early 2011 was linked to an increase in the number of cases of melioidosis in the arid central parts of Australia [6]. *B. pseudomallei* was recovered later from the arid desert environment possibly as a result of intense rainfall in the region causing reactivation of latent endemic *B. pseudomallei*. Notably, increased rates of melioidosis were observed in connection with tropical storms in Taiwan [14–17] and tropical cyclones in the Northern Territory of Australia [12].

Baker and colleagues proposed a concept of *B. pseudomallei* dissemination after heavy rainfall [18, 19], when they observed a cluster of cases around geographic features prone to ground water seepage. A higher direct molecular detection rate was recorded in top soil and water samples collected from seeps following intensive rainfall. It has yet to be determined whether these observations are unique to the geography of the study area in North Queensland. However, this study established that risk of melioidosis did not rely on direct exposure to extreme weather. The role of topography in the risk of melioidosis was also explored by Mu et al., where they noted an increase in the rate of *B. pseudomallei* isolation in locations on expansive plains that provide room for development of high wind speeds and inferred an increased risk of infection [14].

## Climate Change

**Fig. 1:**
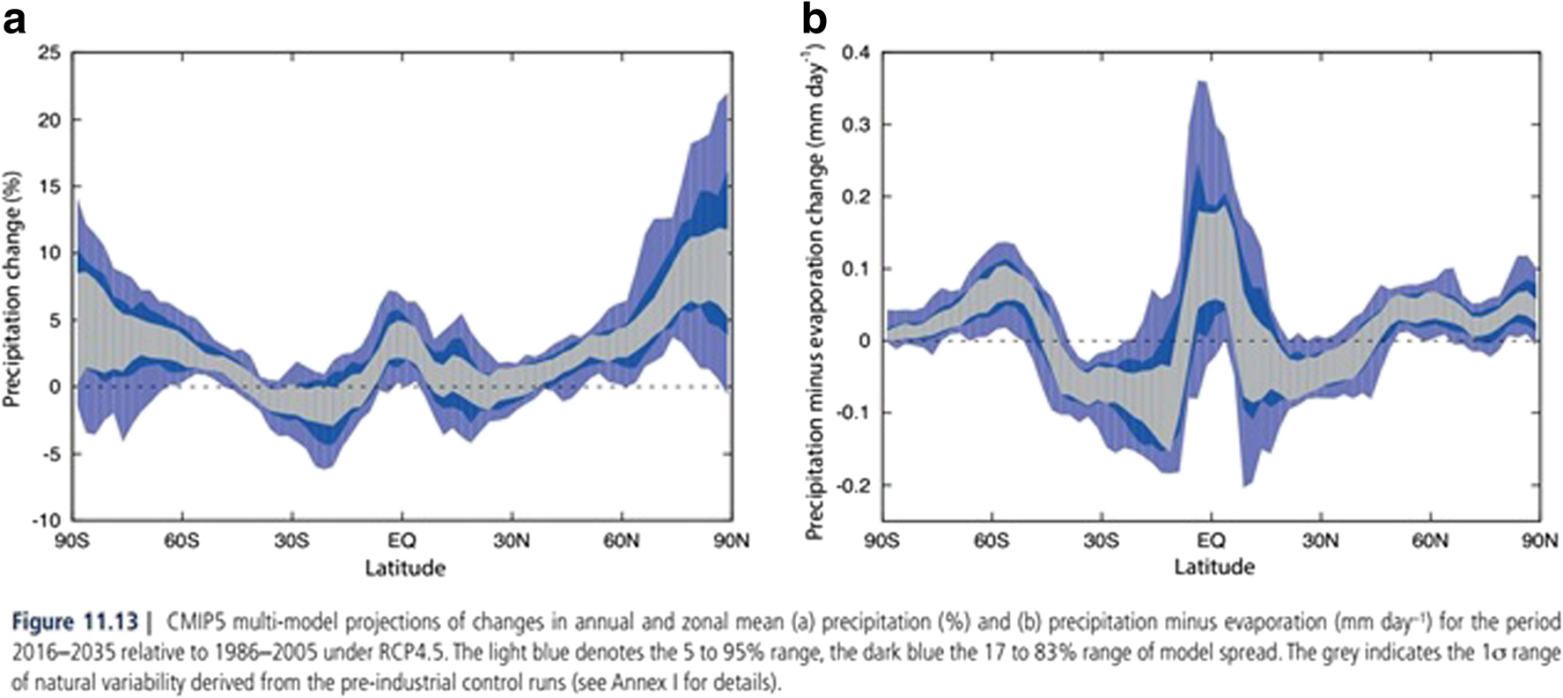
IPCC CMIP5 multi-model near-term (2016–2035) precipitation change by latitude from Climate Change 2013: The Physical Science Basis, Chapter 11 showing a likely decrease in precipitation (**a**) and precipitation minus evaporation (**b**) between 20 and 40 degrees latitude and increases in precipitation outside of those regions [24]

In its simplest form, the impact of global climate change has been equated to global warming and rising sea levels [20••]. For some diseases, these effects may be sufficient to trigger emergence of previously unseen disease [21, 22]. However, in the case of melioidosis, increasing temperatures will not lead to increased cases without other contributing climatic factors, including increases in the amount and severity of rainfall, along with either a latent presence of *B. pseudomallei* in or transport into a new location. Both of these scenarios have already been separately observed [23, 6]. Lastly, susceptible hosts must be present in newly formed hotspots and must be exposed to *B. pseudomallei*. This combination of events may occur as a response to shifting patterns of rainfall. Global precipitation will necessarily increase as a result of greater surface evaporation rates. However, the predicted distribution of changes in precipitation is not even. Near-term multi-model Intergovernmental Panel on Climate Change (IPCC) predictions in annual rainfall suggest a likely decrease in rainfall between 20 and 40 degrees latitude and general increases outside this zone (Fig. 1) [20••, 24]. This means that the already wet tropics and moist mid-latitudes will likely receive more rain at the expense of the mid-latitude subtropical arid and semi-arid areas. More rain will be delivered to all areas by more intense extreme weather events, increasing the opportunities for *B. pseudomallei* exposure [25]. Increases in maximum rainfall in the tropics will further increase melioidosis risk while increased rain outside the tropics will expand the *B. pseudomallei*-receptive regions. Changes in the way we respond to climate change in the existing endemic tropical zone will also play a role in increasing the risk and incidence of melioidosis. More adverse climatic conditions and population growth in established centres will fuel a need for new buildings, establishing new infrastructure and increasing agricultural output [26]. Further drying of the mid-latitude subtropical arid and semi-arid areas will induce a slow process of migration, moving human populations north and south to regions of higher rainfall and further increase the need for intensive agriculture, establishment of new population centres and increased growth and urbanisation [27].

These activities combine to increase our interaction with the environment which is an essential pre-requisite for contracting melioidosis. In Western Australia, the Kimberly and Pilbara regions have seen increasing rainfall and increasing maximum monthly rainfall over the last 60 years [28, 29]. At the same time, agricultural land in the south west of Western Australia has experienced reduced rainfall. This has driven agriculture projects in the Kimberley region, such as the Ord River irrigation scheme, where the generation of Anthrosol soil types may further promote the conversion of melioidosis risk into disease occurrence. Land use was shown to be an important determinant of the presence of *B. pseudomallei* in soil [30]. Anthrosol soil was associated with *B. pseudomallei* presence by Limmathurosakul et al. [31••]. An investigation into the importance of the rhizosphere in the distribution and environmental burden of melioidosis was notably absent from this study and was possibly difficult to model at the 25 km^2^ resolution used. However, an association between roots rich soil and presence of *B. pseudomallei* was described by Kaestli et al. [32]. Importantly this was only observed at undisturbed green-fields sites, while the association was absent at heavily modified sites producing anthrosols. Our demonstration of *B. pseudomallei* and other *Burkholderia* species in association with arbuscular mycorrhizal fungus spores collected from rhizospheric soil samples illustrates the ecological complexity of these microbial habitats [33].

## Localised Climatic Effects on Melioidosis in Western Australia

**Fig. 2:**
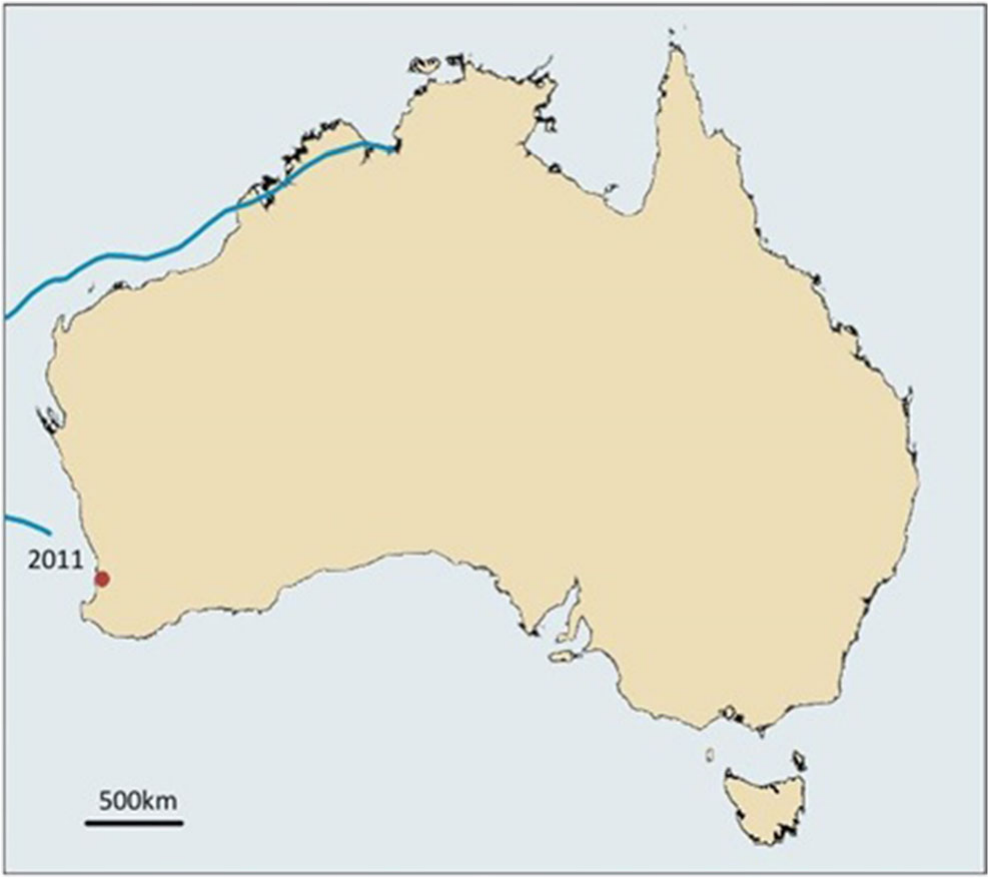
Track of severe tropical cyclone Bianca (2011) and subsequent melioidosis cluster

Climate or weather has been directly implicated in either cases of melioidosis or dissemination of *B. pseudomallei* in three significant events in Western Australia. In January 2012, we investigated a case of cutaneous melioidosis in the City of Mandurah in temperate south-western Australia [34]. The patient had not travelled to northern Australia to provide exposure to what typing indicated was most likely an Australian isolate. Limited environmental sampling in and around the residence did not produce any isolates and the investigation ended. However, starting in December of 2013, a series of cases occurred in the same area. During a joint laboratory and public health investigation, the source was traced to a bottle of saline irrigation fluid in medical care provider shared by all patients and the series of cases ended. While it is likely that the later cases were instigated by exposure to the contaminated saline, the mechanism by which the saline was contaminated was never adequately explained. Having a presumed environmentally acquired infection in the first patient in 2012, we postulate a second environmentally acquired infection attending the same facility most likely provided the necessary material. Typing of the isolates from the case series indicated a near match to an earlier blood culture isolate from Derby in far north-western Australia. A mechanism to link the two areas was needed. An examination of severe weather events preceding the 2012 case identified Severe Tropical Cyclone Bianca as a possible vehicle for transport [35]. This severe weather event started as a low pressure system in near the Western Australia and Northern Territory border on the 21st of January 2011. After moving out into the Gulf of Carpentaria and then over most of the Kimberley coastline, before heading out to sea, the system reached cyclone strength and was named Bianca on the 26th of January (Fig. 2.). Bianca then tracked down the Western Australia coast. While Bianca dissipated over the ocean after the 30th of January, we speculate that something similar to the extra-tropical transition capture mechanism described by Foley and Hanstrum [36], could have moved material kept aloft by the cyclone and deposited it over south-western Australia. Though light, this seeding could have introduced enough contamination for a few sporadic cases. This proposal is speculative and not readily testable. Transport of bacteria over such large distances has not been proven. However, increases in ambient *B. pseudomallei* in tropical storms leading to environmental contamination have been demonstrated as has geographic restriction of cases to defined areas [15–17, 37]. It is perhaps noteworthy that intense weather also in early 2011 in the Northern Territory also led to cases of melioidosis outside of the normal tropical region [6].

Since the 1960s, the Avon Valley to the north east of Perth has produced periodic cases of melioidosis [38•, 39–41]. Primarily veterinary cases, these have all been shown to be caused by a single MLST sequence type [38•]. On the 10th February 2017, a significant storm system produced near record rainfall in the same area [42]. Shortly afterwards an outbreak of systemic infection was reported in a livestock population (N. Buller, personal communication, 21st February, 2017). MLST we performed confirmed the locally endemic sequence type. It was assumed at the time that severe weather was directly responsible for exposure of the animals to *B. pseudomallei*, when the subsequent flooding and erosion seeded the downstream farmland and led to the animal case- cluster. This event validated the hypothesis put forward by Chapple et al., that extreme weather events could reactivate a latent *B. pseudomallei* population and extended the period over which endemicity persisted to at least half a century. Interestingly, the work by Chapple et al., revealed a likely longer period of endemicity extending back before the presence of *B. pseudomallei* was recognised in the area in the 1960s. Earlier transport and delivery by historically regular extra-tropical cyclones is plausible as an explanation for the organism in the area.

Between 2003 and 2007, we conducted an assessment of the occupational risk of melioidosis in an industrial scale mining environment [43]. Over a 7-year period, we conducted environmental sampling for *B. pseudomallei* and melioidosis sero-surveillance in mine site staff and concluded that occupational melioidosis risk was minimal. However, one unusual climate event occurred during this prospective environmental surveillance when in May 2005, a severe weather event occurred that combined high-velocity winds and heavy rain to the Kimberley region where the study site was located. Following this there was a significant increase in mine staff sero-positivity and on the next environmental sampling run, we recovered *B. pseudomallei* with a single PFGE macrorestriction pattern from multiple soil samples near the mine tailings. *B. pseudomallei* with this profile was previously identified in a point source cluster of human cases 500 km to the west [44]. Though laboratory contamination could not be completely excluded as an explanation, the earlier isolate from 1999 was not worked on in 2005. Moreover, bacteria with that PFGE pattern had not been recovered from other environmental samples before the extreme weather event or recovered in studies since. We attributed the arrival of isolates with this PFGE profile on the mine site to the severe weather that immediately preceded it. Subsequent multilocus sequence typing (MLST) showed that the isolates from 1997–1999 and from 2005 were also indistinguishable by this typing system.

**Fig. 3:**
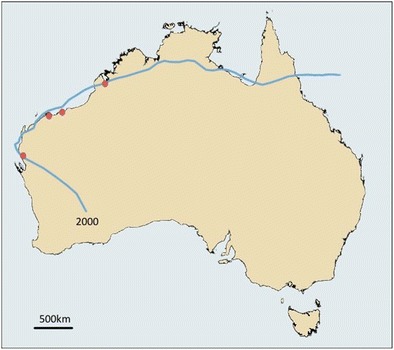
Track of severe tropical cyclone Steve (2000) and subsequent melioidosis cases

**Fig. 4:**
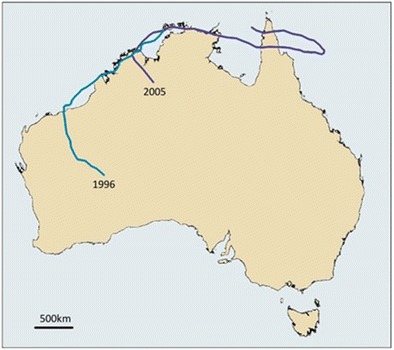
Tracks of severe tropical cyclone Rachel (1996) and tropical cyclone Ingrid (2005)

Tropical cyclones (TC) are complex weather systems that cross the northern Australian coastline between late spring and early autumn. Around 1–5 cyclones cross the north-western Australian coast each year, following a more predictable path that cyclones in other parts of the Indian and Pacific Ocean basins. That relative predictability provides an opportunity to observe the relationship between tropical cyclones and laboratory-confirmed cases of melioidosis. In the decade following the start of melioidosis notification in Western Australia, most of the cases did not occur on or close to a cyclone track, during or in the months after the cyclone season, even though most cases occurred during the tropical wet season. However, eight cases were recorded on cyclone tracks within 3 months of cyclone occurrence. The majority of these followed tropical cyclones that hugged the north-western coastline and which made multiple landfalls (*X* ^2^ = 21.65; *p* < 0.0001). The single most prolific system associated with cases of melioidosis was TC Steve in 2000 (Fig. 3), which travelled anticlockwise over Far North Queensland, the Northern Territory and Western Australia. Four culture-positive cases occurred in its wake, the first only 7 days after TC Steve passed through. These cases were caused by *B. pseudomallei* belonging to different MLST genotypes, arguing against airborne dissemination from a common source. The single apparent long-distance dissemination during this period was the connection between an environmental isolate in the East Kimberly following shortly after TC Ingrid, which arose close to the origin of severe tropical cyclone (STC) Rachel in 1996; a system that provides a plausible explanation for contamination of the aerator tower implicated as the upstream source of the West Kimberley melioidosis outbreak in late 1997 [45, 44]. STC Rachel and TC Ingrid both crossed the Tiwi Islands as well as a point on the Australian mainland close to the remote Kalumburu community that are both endemic for melioidosis (Fig. 4). Either location may have acted as a common source for the specific *B. pseudomallei* sequence type mentioned above that caused fatal septicaemic melioidosis in a remote West Kimberley and was recovered from a mine site 500 km away 8 years later [43, 44]. This once-in-a-decade occurrence in spite of notifiable disease surveillance and prospective genotyping of clinical *B. pseudomallei* isolates indicates the rarity of long distance bacterial transfer as an explanation for septicaemic melioidosis [28]. The predominance of unique *B. pseudomallei* sequences types is more consistent with exposure to locally occurring bacteria that have been present in the soil or surface water for an indeterminate period before each cyclone. The sporadic nature of occasional cases of melioidosis on or around cyclone tracks and following in their immediate aftermath does not allow us to distinguish between exposure to recent rain saturation of the soil, surface water runoff or localised generation of coarse aerosols. However, the paucity of lower respiratory infection as the predominant clinical presentation of severe melioidosis in Western Australia is an argument against exposure to high-density bacterial aerosols [28].

## Conclusion

Given that melioidosis is an infection of environmental encounter, climate-driven modification of the normal habitat of its causal agent, *B. pseudomallei*, has both direct and indirect impact on the geographic distribution and seasonal incidence of melioidosis. Data is lacking on the possible role of climatic features such as rainfall, sunlight, wind and extreme weather events on type and severity of infection. The complex interaction of weather, land surface, its flora and fauna and human population make prediction of climate effects on melioidosis a challenging task. The rare but recurring occurrence of melioidosis in temperate Western Australia raises two questions:

A. Is transport by extreme weather rare?, or
B. Is transport common but bacterial strains that can persist in temperate climates are rare?

It is apparent that the long-term trends emerging from studies in climate change, the physical geography of the melioidosis-endemic zone and disease notification will fuel further studies into the effects of climate on melioidosis.
